# Modulation of the intramedullary pressure responses by calcium dobesilate in a rabbit knee model of osteoarthritis

**DOI:** 10.3109/17453674.2011.618916

**Published:** 2011-11-24

**Authors:** James E Miles, Asger Wenck, Christian Fricker, Eiliv L Svalastoga

**Affiliations:** ^1^Department of Small Animal Clinical Sciences, Faculty of Life Sciences (LIFE), University of Copenhagen, Denmark; ^2^Chaltbrunnstrasse 18, 8165 Schöfflisdorf, Switzerland

## Abstract

**Background and purpose:**

The presence of bone marrow edema in patients with osteoarthritis is associated with pain and disease progression. Management of bone edema with the synthetic prostacyclin iloprost may be complicated by side effects. Calcium dobesilate, a treatment for chronic venous disease, shares some pharmacological actions with iloprost but appears to be better tolerated. Anecdotal reports have suggested that calcium dobesilate may be useful for medical management of osteoarthritis, possibly by reducing bone marrow edema, and this study was performed to investigate possible benefits of treatment.

**Methods:**

The effects of a 6-week period of oral calcium dobesilate administration on tibial intramedullary pressure dynamics and physical joint characteristics were evaluated in 20 rabbits with unilaterally induced knee osteoarthritis that were randomly allocated to either a treatment group or a placebo control group. Treatment or placebo started 8 weeks after induction of osteoarthritis, and was followed by a 4-week washout period.

**Results:**

Calcium dobesilate did not affect joint thickness or range of motion, nor individual pressure measurements, compared to placebo. Pressure ranges in the operated limb were greater than in the intact limb after 8 weeks, and approached those of the intact limb after 6 weeks of treatment with calcium dobesilate but not with placebo. Inter-limb differences were lower (p = 0.02) in the dobesilate group following the washout period.

**Interpretation:**

Calcium dobesilate had a detectable effect on pressure dynamics in the subchondral bone of osteoarthritic joints in this model. The significance of these effects for pain and function should be established.

Osteoarthritis is a common condition causing disability in elderly human and veterinary patients (Vaughan 1990, Felson and Zhang 1998). The precise cause of pain in osteoarthritis is unknown (Felson et al. 2001). Intra-articular anesthesia does not universally produce pain relief in patients with osteoarthritis (Creamer et al. 1996), which suggests that extra-articular sources of pain may be involved.

Patients with knee pain are more likely to have bone marrow edema on MRI than those without pain (Felson et al. 2001), and presence of bone marrow edema is a risk factor for progression of knee osteoarthritis (Felson et al. 2003).

The cause of the edema is unclear. Arnoldi et al. (1980) proposed that impaired venous drainage from the bone marrow could be a cause of pain in osteoarthritis, and increased intramedullary pressures have been reported in the femoral head of humans with osteoarthritic hips (Kiaer et al. 1988). Fricker et al. (1995) have proposed that accumulation of osmotically active proteins in the subchondral bone tissue leads to development of a local compartment syndrome and increased intraosseous pressure in osteoarthritis; resorption of these proteins will lead to resolution of the compartment syndrome and break the cycle of worsening disease.

Tramadol and the synthetic prostacyclin iloprost have provided similar analgesia in patients with painful bone marrow edema of the knee, but regression of edema was found to be more pronounced in the patients receiving iloprost (Mayerhoefer et al. 2007). Improvements in symptoms with iloprost treatment have also been reported for bone marrow edema of the foot (Aigner et al. 2005). The actions of iloprost include inhibition of platelet and leukocyte activation, vasodilatation, and reduction of vessel wall permeability (Grant and Goa 1992). Reported side effects in man include severe headaches, erythema, and nausea (Aigner et al. 2001, Jager et al. 2008).

Calcium dobesilate (calcium 2,5-dihydroxybenzene sulfonate) is a synthetic venoactive drug with multiple effects that include inhibition of serotonin-, bradykinin-, and histamine-induced capillary permeability, inhibition of prostaglandin and thromboxane synthesis (resulting in reduced platelet aggregation and blood viscosity), reduction of experimental lymphedema and intralymphatic pressure, increased lymphatic flow, reduced angiogenesis, and reduced albumin leakage (Tejerina and Ruiz 1998). Macrophage-driven removal of proteins has been described in models of lymphedema (Casley-Smith and Casley-Smith 1985). Many of these actions are comparable to those of iloprost. Oral safety in animals appears high (Tejerina and Ruiz 1998) and in a large study of diabetic patients, no relevant drug-related complications were recorded (Haritoglou et al. 2009). Efficacy in reducing lower limb edema has been shown in patients with chronic venous disease (Flota-Cervera et al. 2008).

Calcium dobesilate treatment of navicular syndrome in horses, characterized by increased intramedullary pressures and edema in the navicular bone (Svalastoga and Smith 1983, Pool et al. 1989), has been reported anecdotally (Fricker 2008). The oral formulation and low toxicity of calcium dobesilate make it an interesting drug for management of bone marrow edema.

This study was designed to investigate the effect of calcium dobesilate treatment on intramedullary pressure in a rabbit model of osteoarthritis.

## Material and methods

The trial population consisted of 20 age-matched female New Zealand White rabbits, housed in groups of 5 in 4m^2^ pens with deep litter and environmental enrichment. The rabbits were 3 months old and weighed 2.4 kg (SD 0.1 kg) at the start of the trial, and they were individually identifiable by means of ear tattoos. Approval for their use in this study was obtained from the Animal Experiment Inspectorate (Dyreforsoegstilsynet, Denmark).

Anesthesia was induced using a combination of intravenous midazolam, fentanyl, and fluanisone, and maintained with isoflurane in oxygen.

Knee joint width was measured using digital calipers with a spring-loaded contact plate (ReDog, Västerås, Sweden), ensuring that all measurements were made with the same pressure. Maximum extension and flexion angles were obtained using a goniometer centered on the stifle joint and aligned with the greater trochanter proximally and the lateral malleolus of the tibia distally. Proximal thigh muscle bulk was evaluated by measuring the circumference of the thigh using a measuring tape with a spring-loaded marker (ReDog) to ensure that tape tension was identical for each measurement.

**Figure 1.**
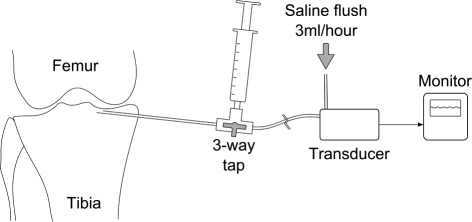
Study instrumentation. This schematic diagram shows the proximally directed 22-gauge spinal needle placed through the medial cortex of the tibia, connected via a 3-way tap to a saline-filled syringe for flushing and a pressure transducer. The pressure transducer, line, and needle were subject to a constant low rate (3 mL/h) flushing with saline to ensure patency.

Following routine aseptic preparation and using the insertion of the medial collateral ligament as a landmark, a 38-mm 22-gauge spinal needle was inserted through the cortex of each tibia, angled slightly proximally, in order to place the tip in the medullary cavity below the tibial plateau (Figure 1). The bevel was oriented proximally in all instances. Once placed, the needle was connected via a 3-way tap and fluid line to a pressure transducer (Gabarith PMSET 1DT-XX; Becton Dickinson) which was connected to a monitor. Following connection, the needle was flushed with a small volume (0.1–0.2 mL) of 10 IU/mL heparinized saline to ensure patency. If there was resistance to injection or no pulsatile waveform was identified, the stylet was replaced and the needle advanced and rotated to free it from any obstruction. A resting value for intramedullary pressure was obtained once the mean pressure reading had stabilized and the needle was flushed with 1 mL of heparinized saline to increase the measured pressure to 200 mmHg or greater. Peak pressure after injection and mean pressure 15 s after injection—and at 30-s intervals for 10 min after completion of injection—were recorded. All needle insertions were performed by one investigator (AW).

Once pressure measurements were completed bilaterally, the right cranial (anterior) cruciate ligament was transected via a lateral mini-arthrotomy, in order to destabilize the joint. All ligaments were intact prior to transaction and no instability was noted in the unoperated knees. Wound closure was routine. All surgeries were performed by one investigator (JM).

Perioperative analgesia was provided by subcutaneous injection of 0.3 mg/kg meloxicam and of 0.03 mg/kg buprenorphine. Postoperatively, meloxicam was administered orally at 0.3 mg/kg once daily for 10 days, and thereafter at 0.1 mg/kg once daily throughout the duration of the study.

All measurements were repeated after 8 weeks, and the rabbits randomized to treatment and placebo control groups. The investigators were blinded regarding which animals were in which groups. Rabbits in the treatment group received calcium dobesilate orally, 25 mg/kg twice daily for 6 weeks, whereas those in the placebo group received lactose at a dose equivalent to that found in the compounded drug.

Measurements were repeated after 6 weeks. The calcium dobesilate and lactose treatments were withdrawn. After another 4 weeks, final measurements were taken and the rabbits were killed.

### Statistics

Statistical testing between groups and stages was performed using mixed-model ANOVA with SAS 9.1 software, and a Bonferroni correction for multiple comparisons was applied. Statistical significance was set at p < 0.05.

## Results

Few complications occurred. 1 rabbit died during induction of anesthesia at the start of the study, and was replaced by another age-matched rabbit. 2 rabbits in the placebo group developed a seroma at the operation site, both of which resolved spontaneously within a week. An additional needle had to be placed in 3 limbs (2 at the pre-treatment stage and 1 at the post-treatment stage) due to failure to obtain a satisfactory placement with the first. All needles were patent on removal. All joints were unstable after surgery using the Lachman test.

Pressure data were examined to find the minimum (from 15 s to 150 s) and maximum (from 180 s to 600 s) pressure values, the mean pressure value (from 180 s to 600 s) and the baseline pressure value prior to saline injection. The time cut-off points were selected to avoid inclusion of high readings from the initial saline injection in the means and maxima. Differences in pressure measurements between intact and operated limbs within each group were compared. No statistically significant differences in the minimum, maximum, mean, or baseline pressure values were observed between treatment groups at any stage.

**Figure 2.**
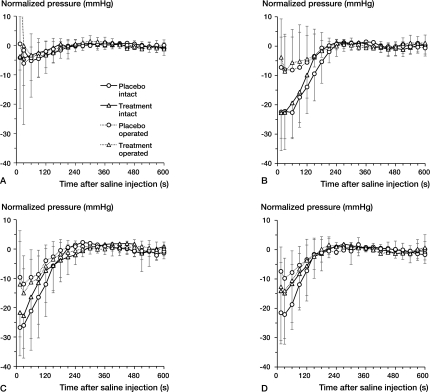
Mean-normalized pressure following saline injection for operated and intact limbs in the treatment and placebo groups at the four measurement stages: at induction of arthritis (A), prior to treatment/placebo (B), following treatment/placebo (C), and after washout period (D). Pressure values were individually normalized to the mean value for the time period 180–600 s to simplify comparisons of pressure changes between groups. Although mean pressures for all limbs are initially similar, the operated and intact limbs diverge with the development of arthritis. This divergence diminishes markedly following treatment with dobesilate, but barely changes in the placebo group. Error bars represent 1 SD.

**Figure 3.**
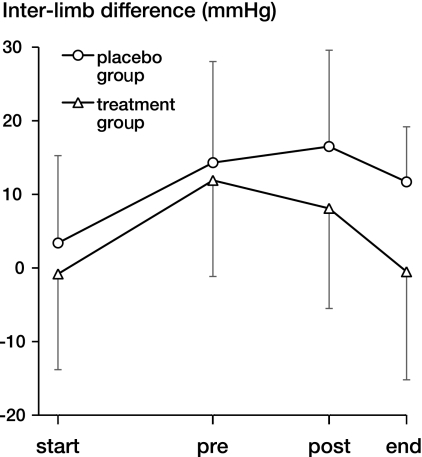
Pressure range differences between intact and operated limbs for the treatment and placebo groups throughout the study period (start: start of study; pre: pre-treatment; post: post-treatment; end: end of study). The pressure range was defined as the difference in measured pressure between the minimum over 15–150 s post-injection and the mean over 180–600 s post-injection. Differences were calculated as intact range minus operated range and displayed as mean values. Error bars represent 1 SD and are shown on one side only for clarity.

Pressure data were normalized to the mean value found above for each stage of the study (Figure 2). Whilst curve characteristics were similar for both groups and both limbs at the start of the study, the intact and operated limb curves diverged in the first 180 s after saline injection by 8 weeks after induction of arthritis (p = 0.01). This divergence became less in the treatment group by the end of the treatment period and became further reduced during the washout period. As a measure of this divergence, pressure ranges were calculated between the minimum pressure in the time period 15–150 s and the mean pressure in the time period 180–600 s for each limb. Differences in this pressure range between operated and intact limbs were calculated for each rabbit by subtraction of the range figure for the operated limb from that of the intact limb (Figure 3). No statistically significant differences were found in the pressure range differences between the treatment and placebo groups for the first three stages of the study, but the pressure range difference was significantly different between groups at the end of the study (95% CI: 1.9–22 mmHg; p = 0.02).

Changes in joint width, maximum extension or flexion, range of motion, and thigh circumference were similar for the treatment and placebo groups. Compared to the intact stifle, joint width increased statistically significantly, and joint maximum extension, flexion, and range of motion decreased significantly over the study period in the operated stifles of both the treatment and placebo groups. A difference was found in the thigh circumference difference between intact and operated limbs between the end of treatment and the end stages of the study (95% CI: 0.1–0.9 cm; p = 0.005) in the placebo group but not in the treatment group.

No statistically significant differences in body weight or change in body weight were found between the treatment and placebo groups, but differences were found between all stages of the study (p < 0.001).

## Discussion

Section of the cranial (anterior) cruciate ligament in rabbits reliably induces osteoarthritic changes by 8 weeks (Yoshioka et al. 1996), and a rabbit model has been validated previously for evaluation of oral medications (Tiraloche et al. 2005).

Our physical findings are in broad agreement with a previous study of monoarticular arthritis in rabbits (Pitt and Lewis 1984), and with clinical findings in dogs (Piermattei et al. 2006). Changes in joint width and maximal extension and flexion angles were not affected by treatment with calcium dobesilate. Although we expected to find a general reduction in thigh circumference in the operated limb relative to the intact limb, this was not confirmed. During the study, we felt that the use of the measuring tape was not particularly accurate, due to difficulties retaining the tape at the correct height on the limb, obstruction of the tape by fur, and subjectivity in positioning.

Previous investigation of intramedullary pressure in the rabbit gave a wide range of normal values for the resting pressure (Thomas et al. 1982): similarly, we found wide ranges for all single pressure measurements (baseline, mean, minimum, maximum), which prevented meaningful comparison of the raw data. Intramedullary pressures are linearly related to systemic arterial pressures (Kiaer et al. 1990) and it may be that individual responses to the anesthesia regime used contributed to this wide variation. Analysis of pressure range (from minimum to mean pressure) yielded interesting results. Initially, the pressure range was small but it increased dramatically in the intact limbs, and moderately in the operated limbs, by 8 weeks. The cause of this change is unclear. Possible factors include meloxicam treatment, the effect of placing a spinal needle through the cortex, altered limb loading as a result of osteoarthritis, and growth-related changes. The most plausible reasons for the difference in effect size between the intact and operated limbs are altered weight bearing or intramedullary edema.

Following the treatment period, the pressure ranges of the treated limbs became more similar—which could indicate a lessening of intramedullary edema or improved weight sharing between the limbs. In contrast, the difference between the hind limbs of the placebo group was exacerbated: the osteoarthritis could reasonably be expected to have worsened further during this period (Yoshioka et al. 1996). Unexpectedly, this trend in the treatment limbs continued during the washout period at the end of the study. The placebo limbs also showed some improvement. This could be due to periarticular thickening achieving stabilization of the knee joint, and thus an improvement in limb function. The further improvement in the treatment limbs may therefore represent a combination of acquired joint stability and improvements due to prior dobesilate treatment. Perseverance of the drug is unlikely because elimination of calcium dobesilate after oral administration is 50% at 24 h (Tejerina and Ruiz 1998). Interestingly, in a study of chronic venous disease in humans it was noted that there was a persistent positive effect of dobesilate on quality of life compared to placebo (Martinez-Zapata et al. 2008), suggesting a sustained therapeutic action.

Calcium dobesilate could have a positive effect in bone marrow edema and osteoarthritis through several mechanisms. Since normal bone lacks lymphatic tissue (Edwards et al. 2008), the positive effect of calcium dobesilate on lymphatic circulation (Casley-Smith 1985) is unlikely to contribute. Calcium dobesilate has been shown to reduce swelling and protein concentrations in edema by a mechanism that is independent of lymphatic drainage, probably by a macrophage-mediated effect (Casley-Smith and Casley-Smith 1985). Moreover, by reducing capillary permeability and albumin leakage (Tejerina and Ruiz 1998), the tendency for edema formation is diminished. In addition, reduction of blood viscosity and improvements in blood flow (Tejerina and Ruiz 1998) may aid in reducing the hypoxia that occurs in subchondral bone (Kofoed 1986, Kiaer et al. 1988), in the synovial fluid (Kofoed 1986), and in the articular cartilage—as evidenced by increased hypoxia-inducible factor activity (Pfander et al. 2005).

Alterations in growth rate or weight gain can be used as an objective means of monitoring the efficacy of analgesics in laboratory animals (Brennan et al. 2009). It does not appear that calcium dobesilate administration resulted in an enhanced analgesic effect over and above that of meloxicam, since no differences in weight between groups were identified.

In summary, this study indicates that calcium dobesilate has an effect on intramedullary pressure adjacent to an osteoarthritic joint. The clinical significance of this in terms of pain management cannot be quantified using this model. These conclusions should be seen in light of the small size of the study, and further investigation using larger groups is recommended. Although we did not measure the presence of edema directly, it can be inferred from our results that calcium dobesilate has an effect on the presence and severity of subchondral bone edema in osteoarthritic joints. Use of MRI to identify the presence or absence of edema, or a method of quantifying limb use (such as force plate analysis) would be valuable in determining what the effects of calcium dobesilate are, and in defining a potential role for this compound.
